# Supplementary material for: Moderation by better sleep of the association among childhood maltreatment, neuroticism, and depressive symptoms in the adult volunteers: A moderated mediation model
Source: PLoS One. 2024 Jul 12;19(7):e0305033. doi: 10.1371/journal.pone.0305033 (PMC11244762; doi:10.1371/journal.pone.0305033)
Supplement: Attachment — Submitted filename: [PONE-D-24222]_Response to Reviewers_28_Apr_2024.docx [file pone.0305033.s001.docx]

April 28, 2024

**Dr. Anthony A. Olashore, MBCHB, PhD, FWACP**

Academic Editor

*PLOS ONE*

RE: **“Moderation by better sleep of the association among childhood maltreatment, neuroticism, and depressive symptoms in adult volunteers: a moderated mediation model”** (submission ID no.: PONE-D-23-24222)

Dear Dr. Olashore,

We thank you as well as the reviewers for the thoughtful comments regarding our above manuscript (submission ID no.: PONE-D-23-24222), dated July 31, 2023. We have carefully reviewed the comments and provided details of our corrections, as below, which we hope will meet your approval. The English language in the revised manuscript has been rechecked by a native English speaker experienced in proofreading scientific documents. The revised manuscript is also attached.

We believe that the revised manuscript is a more balanced representation of our work, and hope that it is now acceptable for publication in *PLOS ONE*.

Sincerely yours,

**Jiro Masuya, MD, PhD**

Associate Professor

Department of Psychiatry

Tokyo Medical University

6-7-1 Nishishinjuku, Shinjuku-ku, Tokyo 160-0023, Japan

Phone: +81-3-3342-6111

Fax: +81-3-3340-4499

E-mail: j-masuya@tokyo-med.ac.jp

**POINT-BY-POINT RESPONSES TO THE REVIEWERS’ COMMENTS**

We thank the reviewers for the helpful advice and comments, which have enabled us to substantially improve our manuscript.

**REVIEWER #1**

**1.  Please go through the manuscript and revise grammar. Some sentences are too long.**

Response:

We thank you for your comments. In accordance with your suggestion, we have revised some sentences and asked a native English speaker experienced in proofreading scientific documents to recheck the paper.

**2. In my opinion, the authors should acknowledge limitations of using self-administered instruments in their limitations**

**3. As mentioned under limitations, sleep disturbance is a well researched and documented symptom of depression; there is however evidence suggesting it as a risk factor as well. The authors should justify why in this study they chose to consider it as a risk factor only and not account for it as a symptom signifying severity of depression.**

Response:

We thank you for your comments. In accordance with the comment, we have revised the limitations section of the revised manuscript, as follows.

“Third, subjects in this study were not confirmed to have depression or other psychiatric disorders. In addition, we did not investigate the presence or absence of an actual diagnosis of depression in the patients, as only a self-administered survey was used in this study. Therefore, the results of this study cannot be used as being associated with depression.” (page 19, lines 277 to 281)

“Sleep disturbances are a well-known symptom of depression and a factor indicating the severity of depression. However, in the present study, we did not consider sleep disturbances as only a symptom of depression, but also as a risk factor for enhancing the effects of CM on depressive symptoms in adulthood, and the effects of neuroticism on depressive symptoms in adulthood [32,33].” (page 19, lines 281 to 285)

**REVIEWER #2**

**1.  The authors mentioned the mean age of participants; however, I suggest that authors include the sociodemographic characteristics of participants (add a table summarising this). This will help readers when they want to make decisions in evidence-based medicine. They can compare their clients or patients with the study population for similarity (external validity of the paper).**

Response:

We thank you for your comments. In accordance with your suggestion, we added a table with the demographic information of the 584 subjects, as Table 1 of the revised manuscript. (page 12, lines 173 to 174)

**2. Maybe the authors could also add non-probability sampling as a limitation of the study.**

Response:

We thank you for your comments. In accordance with the comments, we have revised the limitations section, as follows.

“First, the subjects in this study were recruited using convenience sampling, which is not strict probability sampling. This reduces the randomness of subject selection, which may reduce the accuracy of the results by including potential bias.” (page 19, lines 271 to 274)
